# Urinary Tract Infections and Preeclampsia among Pregnant Women Attending Two Hospitals in Mwanza City, Tanzania: A 1:2 Matched Case-Control Study

**DOI:** 10.1155/2019/3937812

**Published:** 2019-03-27

**Authors:** Joshua Kaduma, Jeremiah Seni, Clotilda Chuma, Richard Kirita, Fridolin Mujuni, Martha F. Mushi, Frank van der Meer, Stephen E. Mshana

**Affiliations:** ^1^Department of Obstetrics and Gynaecology, Bugando Medical Centre and Catholic University of Health and Allied Sciences, Mwanza, Tanzania; ^2^Department of Microbiology and Immunology, Weill-Bugando School of Medicine, Catholic University of Health and Allied Sciences, Mwanza, Tanzania; ^3^Department of Obstetrics and Gynaecology, Sekou Toure Regional Referral Hospital, Mwanza, Tanzania; ^4^Faculty of Veterinary Medicine: Ecosystem and Public Health, University of Calgary, Calgary, AB, Canada

## Abstract

Urinary tract infection (UTI) and preeclampsia are common among pregnant women and are associated with adverse maternal-fetal and neonatal outcomes. Despite this, limited information exists on the association between UTIs and preeclampsia in Tanzania to guide specific management and thereby averting the adverse outcomes. A 1:2 matched case-control study (by age and gravidity) involving 131 pregnant women with preeclampsia (cases) and 262 without preeclampsia (controls) was conducted. Sociodemographic and clinical information was collected using a questionnaire. Midstream urine samples were collected during admission for culture and antimicrobial susceptibility testing (AST). Out of 393 pregnant women enrolled, 110 (28.0%), 95% CI: 23.8%-32.7%, had significant bacteriuria [cases: 50.4% (66/131) and control: 16.8% (44/262)]. Pregnant women with preeclampsia had 7.7 odds of having significant bacteriuria than those without preeclampsia [OR=7.7, 95% CI (4.11-14.49); p-value <0.001].* Escherichia coli*, 50 (45.5%), and* Klebsiella *spp., 25 (23.6%), predominated, and resistance to gentamicin, ceftriaxone, and piperacillin-tazobactam ranged from 9.0% to 29.0% in these dominant species. Extended spectrum beta lactamases (ESBL) production in* Escherichia coli* and* Klebsiella* spp. was 18.0% (9/50) and 15.4% (4/26), respectively. Routine urine culture and AST among pregnant women with preeclampsia should be introduced in the antenatal clinics to ensure prompt management. Delineation of maternal-fetal and neonatal outcomes among pregnant women with preeclampsia and UTIs would be of interest in future studies.

## 1. Introduction

Pregnancy is a state associated with physiological, structural, and functional urinary tract changes which most often promote ascending of pathogens into the urinary bladder resulting into urinary tract infections (UTIs) [1]. The magnitude of UTIs among pregnant women is higher than other healthy women in the general population [2]. Both symptomatic and asymptomatic UTIs are common among pregnant women and are associated with adverse outcomes to mothers and their newborns, which include (but not limited to) pyelonephritis, urosepsis, premature labor, and still births [1, 3–5].

Preeclampsia is a condition in which there is onset of high blood pressure (> 140/90 mmHg) in the second half of pregnancy (i.e., >20 weeks gestation age) and proteinuria [6]. It is associated with high maternal-fetal and newborns morbidity and mortality [6]. Preeclampsia pathogenesis is still not well elucidated but existing reports indicate that it is due to abnormal placentation resulting into abnormal perfusion, and hence release of inflammatory factors like cytokines which result into endothelial dysfunction [7–9]. Strikingly, this pathogenesis is most often associated with infections, including UTIs [7, 10, 11]. Existing data, although from few studies, are compelling that there is a potential association between preeclampsia and UTIs, even after adjusting for potential confounders [10, 12–14]. Moreover, pregnant women with preeclampsia are at increased risk of developing adverse maternal-fetal and neonatal outcomes as reported from WHO secondary analysis in low and middle income countries (LMICs) [10]. Similarly, in Tanzania, the incidence of preeclampsia at Bugando Medical Centre (BMC) was reported to be 1.4%, with maternal and perinatal case fatality rates being 7.9% and 20.7%, respectively [15]. However, the study at BMC did not delineate the potential contribution of UTIs as previously reported in other studies [7, 15].

Therefore, in the light of high UTIs among pregnant women in Tanzania [5, 16, 17], and the ongoing effort to combat the dual burden of communicable and noncommunicable diseases in LMICs [18, 19], this study was conducted to evaluate the association of preeclampsia and UTIs so as to guide specific management.

## 2. Materials and Methods

### 2.1. Study Design and Settings

This was a hospital based 1:2 matched case-control study conducted for 3 months from March 2017 to May 2017 at BMC and Sekou Toure Regional Referral Hospital (SRRH). BMC is a tertiary hospital in the North-western part of Tanzania. It serves a catchment population of 16 million people; it has 950 beds and 78 obstetrics ward beds, with approximately 750 deliveries per months. SRRH is a regional referral hospital in the North-western part of Tanzania. It serves a catchment population of 2.7 million people; it has 375 beds and 49 obstetrics ward beds, with approximately 700 deliveries per months.

### 2.2. Study Population

Pregnant women at gestation age >20 weeks with preeclampsia (cases) and without preeclampsia (control) attending antenatal clinics, as well as those admitted in antenatal and labor wards within 24 hours at BMC and SRRH were enrolled. One case was matched to two controls by age and gravidity, and all eligible participants were admitted on the same day and relevant clinical samples taken. Preeclampsia was defined as blood pressure of more than 135/90 mmHg in the second half of pregnancy (i.e., >20 weeks gestation age) accompanied with proteinuria [6]. Severe preeclampsia was defined as high blood pressure of more than 155/105 mmHg accompanied with proteinuria. Proteinuria was defined as the presence of at least 30 mg of protein in one decilitre of urine (which is equivalent to at least +1 proteinuria) using dipsticks (Mission® Urinalysis Reagent Strips, ACON Laboratories Inc., CA, USA). The blood pressure was measured two times with an interval of three hours by resident doctors or obstetricians before the patient is enrolled into the study, using Aneroid sphygmomanometer Lian Nano CE 0469®, Spengler, France. Pregnant women with multiple gestations, renal disease, chronic hypertension, diabetes mellitus, premature rupture of membrane, and dental infections were excluded from the study.

### 2.3. Sample Size Estimation

Sample size was calculated using a “power” command in STATA software version 13 (Stata Corp, Texas, USA) which includes Fleiss considerations. We assumed 1:2 recruitment strategy of cases to controls, with a prevalence of bacteriuria among cases and controls to be 19% and 3%, respectively [20]. Although the minimum number required to detect a 16% difference among the groups was only 167 (56 cases and 111 controls) at a 90% power, during the study period we were able to recruit 131 cases and 262 controls.

### 2.4. Data Collection and Laboratory Procedures

A structured pretested questionnaire was used to collect sociodemographic and clinical information. Swahili language was used during interview; indigenous languages were used when necessary in respective participants. During admission, participants were instructed by trained nurses/obstetricians/investigators on how to collect midstream urine sample using sterile plastic urine containers after cleaning their hands and perineum with water (front to back direction) in a wash room [21]. Urine samples were sent to the laboratory and processed within two hours of collection and if delay was anticipated, samples were placed into a cold box at 4°C and processed within 4 hours after collection.

Urine specimens were inoculated in MacConkey agar and blood agar plates (OXOID, UK) and incubated at 35-37°C for 18-24 hours. Interpretation of bacteria growth was based on bacterial colony forming unit (CFU) per milliliter of urine, and the significant bacteriuria was defined as >10^5^ CFU/ml of midstream urine [5, 22]. Identification of Gram positive and Gram negative bacteria was done by using biochemical tests as previously described [21]. Antimicrobial susceptibility testing (AST) of the isolates was done using conventional disc diffusion method following the Clinical Laboratory Standard Institute (CLSI) guidelines [23].* Escherichia coli *ATCC 259922 and* Staphylococcus aureus* ATCC 25923 reference strains were used for quality control of culture and AST.

### 2.5. Data Management

Data were entered into excel sheet and then exported to the STATA software version 13 (Stata Corp, Texas USA) for analysis according to the objectives of the study. Categorical variables were summarized into proportions and compared using Pearson chi2 test (or 1-sided Fisher's exact where appropriate), while continuous variables were summarized using means ± standard deviations or medians (interquartile ranges) depending on the distribution of data. Conditional regression analysis was done to ascertain the association between preeclampsia and other variables (including significant bacteriuria) using odds ratio and their respective 95% confidence intervals. A p-value of less than 0.05 was used as a cut-off point to show the significant association between preeclampsia and variables among pregnant women.

### 2.6. Ethical Considerations

The study clearance was sought and approved by the joint CUHAS/BMC Research Ethics and Review Committee (CREC/173/2017). Permission was also obtained from the Departments of Obstetrics and Gynecology at BMC and SRRH. Written informed consent was requested from the participants after explaining the aim and importance the study, as well as informing participants that their involvement was entirely voluntary. They were further informed that even if they choose not to participate in the study, they would be still be entitled to the standard treatment provided to all pregnant women at BMC and SRRH. For women aged below 18 years, consent was sought from the parent/guardian and they were voluntarily requested to assent for the study. Pregnant women with preeclampsia and those with significant bacteriuria were treated based on standard hospital guidelines.

## 3. Results

### 3.1. Sociodemographic Characteristics of the Study Population

A total of 393 pregnant women aged 16 to 42 years were enrolled in this study, their median age was 25 (21-32) years. Out 393, 131 were cases and 262 were controls. The majority were urban residents 346 (88.0%), married 349 (88.8%), and Christians 302 (76.8%) (Table 1). Of these, 162 (41.2%) and 231(58.8%) were from BMC and SRRH, respectively.

### 3.2. Clinical and Obstetric Characteristics of the Study Participants

Most women were in their third trimester, 385 (98%). The proportion of HIV seropositivity among pregnant women was 1.0%. The mean systolic and diastolic blood pressure at booking for cases were 115.0 ± 11.5 mmHg and 71.3 ± 9.0 mmHg, respectively, while the respective current values were 160.5 ± 16.2 mmHg and 104.6 ± 11.0 mmHg. On the other hand, the mean systolic and diastolic blood pressure at booking for controls were 105.9 ± 10.6 mmHg and 68.2 ± 7.6 mmHg, respectively, while the respective current values were 116.0 ± 10.6 mmHg and 72.1 ± 9.3 mmHg.

A total of 15 (3.8%) pregnant women had clinical symptoms suggestive of UTIs, eleven from cases and four from control group (Table 2).

### 3.3. The Association between Significant Bacteriuria and Preeclampsia

**Figure 1:**
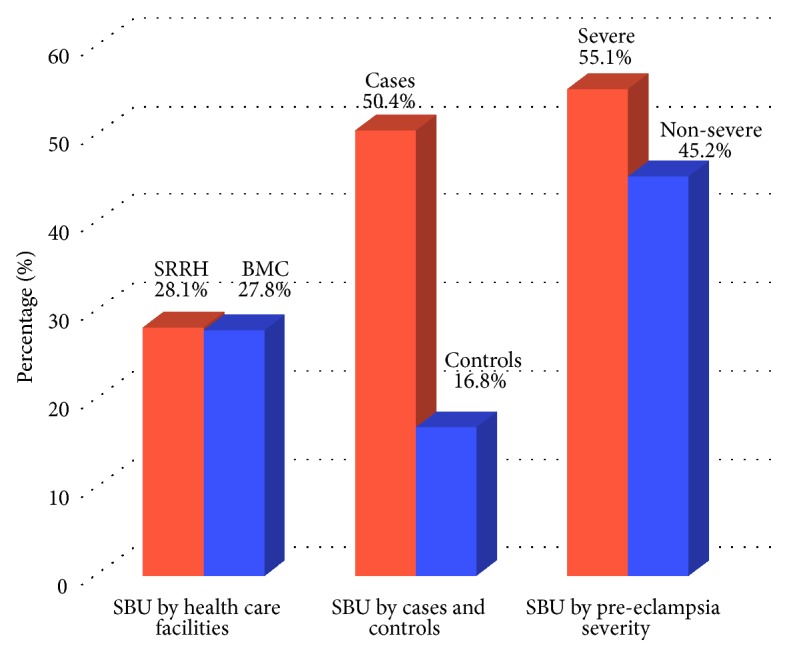
*The distribution of pregnant women with significant bacteriuria by health care facilities, preeclampsia, and severity of preeclampsia*. BMC: Bugando Medical Centre, SRRH: Sekou Toure Regional Referral Hospital, and SBU: significant bacteriuria.

The overall proportion of pregnant women with significant bacteriuria was 28.0% (110/393), 95% CI: 23.8%-32.7%. The proportions of significant bacteriuria among pregnant women attending BMC and SRRH were 27.8% (45/162) and 28.1% (65/231), respectively (Pearson chi2= 0.0061; p-value= 0.938) (Figure 1). Also, significant bacteriuria was found in 27.3% (103/378) of asymptomatic pregnant women compared to 46.7% (7/15) of symptomatic pregnant women (Pearson chi2= 2.699; p-value= 0.100). The proportions of significant bacteriuria among cases and controls were 50.4% (66/131) and 16.8% (44/262), respectively (Pearson chi2= 48.882, p-value <0.001). Moreover, the mean current blood pressure among pregnant women with and without significant bacteriuria was 143.4/92.5 ± 26.3/17.5 mmHg and 125.9/79.2 ± 22.0/17.1 mmHg, respectively.

Pregnant women with severe preeclampsia accounted for 52.7% (69/131), while those with nonsevere preeclampsia accounted for 47.3% (62/131). The proportions of significant bacteriuria among pregnant women with severe preeclampsia were higher than those with nonsevere preeclampsia, although the difference was not statistically significant [55.1% (38/69) versus 45.2% (28/62), Pearson chi2 = 1.2832, p-value = 0.257 (Figure 1)]. Pregnant women with preeclampsia had 7.7 odds of getting significant bacteriuria when compared to those without preeclampsia [OR=7.7, 95% CI (4.11 - 14.49); p-value <0.001]. Factors such as place of residence (rural versus urban), level of education, occupation, religion, and parity were not associated with preeclampsia among cases when compared with controls. Nevertheless, the median gestation age was significantly lower among cases [36 (33-38) weeks] compared to control [39 (37-39) weeks], with the association between lower gestation age among cases being protective [OR=0.73, 95% CI (0.67-0.80); p-value<0.001] (Table 3).

### 3.4. Bacterial Species and Antimicrobial Resistance Patterns among Pregnant Women with Significant Bacteriuria

The predominant bacteria species isolated from 110 pregnant women with significant bacteriuria were* Escherichia coli* 50 (45.5%) followed by* Klebsiella *spp. 26 (23.6%) and* Acinetobacter *spp. 11 (10.0%) (Table 4).* Escherichia coli*,* Klebsiella *spp., and other Gram negative Enterobacteriaceae were predominantly among cases than controls, whereas* Acinetobacter* spp. predominated in controls than cases (Table 4). Over three quarters of Gram positive and negative bacteria were resistant to ampicillin and trimethoprim-sulfamethoxazole. Antimicrobial resistance among* Escherichia coli* and* Klebsiella *spp. isolates to gentamicin, ceftriaxone and piperacillin-tazobactam was less than 30.0%. ESBL production in* Escherichia coli* was 18.0% (9/50) [19.4% (6/31) in cases versus 15.8% (3/19), p-value = 0.532 (based on 1-sided Fisher's exact)], while in* Klebsiella* spp., ESBL production was found in 15.4% (4/26), [20.0% (3/15) in cases versus 9.1% (1/11), p-value = 0.426 (based on 1-sided Fisher's exact]. All Gram negative bacteria were sensitive to imipenem except one* Klebsiella pneumoniae* isolate from a pregnant woman with preeclampsia. Two* Pseudomonas aeruginosa* isolates were all sensitive to gentamicin, ciprofloxacin, piperacillin-tazobactam, and imipenem.* Streptococcus* spp. (n=5) were resistant to gentamicin (20.0%), ciprofloxacin (20.0%), erythromycin (40.0%), and vancomycin (0.0%), (Table 4).

## 4. Discussion

The overall prevalence of significant bacteriuria in this study (16.8%) among pregnant women without preeclampsia is within 13% to 21% range reported a decade ago in Mwanza and Dar es Salaam, Tanzania, among pregnant women in the general population [5, 16]. Of note, the prevalence of significant bacteriuria among pregnant women with preeclampsia was alarmingly high (50.4%), connoting preponderance of UTIs in this vulnerable condition. Similarly, a previous study showed that significant bacteriuria was significantly higher in pregnant women with preeclampsia (19%) compared to those without preeclampsia (3% to 6%) [20]. In the present study, it was also found that significant bacteriuria was approximately twice in symptomatic than asymptomatic pregnant women. The proportions of significant bacteriuria reported in the current study and three other previous studies in Tanzania were higher than 1-9% reported among asymptomatic healthy adult women [2, 5, 16, 17]. This shows that pregnant women with underlying risky conditions like preeclampsia and HIV/AIDS are at increased chances of developing UTIs as opposed to the general healthy women population.

The mean arterial pressure among preeclamptic women was higher in our study (160.5/104.6 ± 16.2/11.0 mmHg) and another study in Brazil (170/110 mmHg), compared to previous studies in Iran 148.2/ 96.1 ± 10.8/5.7 mmHg [8, 24]. Although the reason underlying these differences could not be delineated in these three studies, it may probably be related to variation in factors such as ethnicity, geographical locations or preexisting comorbidities as reported in a study from Australia [25]. We also found higher mean current blood pressure among pregnant women with significant bacteriuria (143.4/92.5 ± 26.3/17.5 mmHg) than among pregnant women without significant bacteriuria (125.9/79.2 ± 22.0/17.1 mmHg).

The link between preeclampsia and immunopathophysiology is still limited, but a report from Brazil and one review have shown the potential linkage between preeclampsia with inflammatory cytokines such as IL-6, IL-8, and INF- *γ*, as opposed to the regulatory cytokines such as IL-4, IL-5, and IL-10 which predominate in normotensive pregnant women [8, 9, 13]. Indeed this proinflammatory state is exemplified by infectious diseases, including UTIs complicating further preeclampsia [7]. Similar to previous studies in the USA, UK, and LMICs [10, 13, 14], the odds of developing significant bacteriuria in pregnant women with preeclampsia were 7.7 among cases compared to controls in the current study. These findings are similar to previous studies in the USA (odds ratio: 3.1), Israel (odds ratio: 1.3 in mild preeclampsia and 1.8 on severe preeclampsia), UK (odds ratio: 1.22), and Iran (odds ratio: 6.8) and from a systematic review from LMICs (odds ratio: 1.13) [10, 12–14, 24]. In our study, we also found high proportion of significant bacteriuria in pregnant women with severe preeclampsia compared to those with nonsevere preeclampsia connoting increased risk of significant bacteriuria with preeclampsia severity, similar to previous studies in Iran and Israel [12, 26]. Therefore, these findings across various countries suffice to support the hypothesis that there is an association between UTIs and preeclampsia and as a matter of fact a routine screening of pregnant women with preeclampsia for UTIs should be strengthened to timely identify them, provide specific management and therefore avert maternal-fetal complications and deaths attributable to the two diseases. It should also be reiterated that previous reviews have reported increase in the risk of preeclampsia among pregnant women with UTIs and periodontal diseases; but with some contradicting association with other bacterial diseases like* Chlamydia pneumonia* and* Helicobacter pylo*ri; viral infections like cytomegalovirus and HIV; and parasitic infections with* Plasmodium falciparum* [7, 27]. Contrary to these findings, a study in South Australia concluded that UTIs is not a risk factor for preeclampsia and the authors associated preeclampsia with ethnicity and preexisting comorbidities like diabetes mellitus [25]. Nevertheless, regardless of the causal relationship in terms of which disease preceded another, coexistence of UTIs and preeclampsia need to be timely diagnosed and properly managed to ensure favorable maternal-fetal and neonatal outcomes [10, 19]. All other factors in this study showed no association with preeclampsia except a protective effect among cases with lower median gestation age. This association may be coincidental or may relate to other factors not studied and therefore not delineated in the present study.

The finding of high antimicrobial resistance to the cheap and readily available antibiotics such as ampicillin and trimethoprim-sulfamethoxazole has been previously reported in Tanzania [5, 16, 17]. Resistance to gentamicin, ceftriaxone, and piperacillin-tazobactam in the current study among dominant bacterial species was below 30%, with ESBL production confirmed in approximately 15% to 18% of these bacterial species. Of note, ESBL production was relatively higher among cases than controls for* Escherichia coli* (19.4% versus 15.8%) and* Klebsiella* spp. (20.0% versus 9.1%), although the differences were not statistically significant, certainly due to the small number of bacterial isolates involved. High ESBL production is worrisome and may be related to the underlying preeclampsia, a condition which may expose these women to repeated admissions, exposure to antibiotics, and sometimes urinary catheterization, which are known to be potential risk factors for multidrug resistance bacteria causing UTIs [28].

## 5. Conclusions

There is strong association between preeclampsia and significant bacteriuria among pregnant women attending BMC and SRRH. Routine urine culture and AST among pregnant women with preeclampsia should be introduced in these two hospitals so as to guide specific antimicrobial therapies. Future studies should be focused on delineation of maternal-fetal and neonatal outcomes, as well as cost-effective interventional measures of managing the two diseases.

## 6. Study Limitations

We did not analyze the proinflammatory cytokines/markers related to abnormal placentation in patients with preeclampsia versus proinflammatory cytokines resulting from hosts' immune response to bacterial agents causing UTIs, and therefore, future studies can ascertain these aspects.

## Figures and Tables

**Table 1:** Sociodemographic characteristics of pregnant women enrolled.

Patient characteristics	Cases, number (%)	Controls, number (%)	All, number (%)
*Residence*			
Urban	118 (90.1)	228 (87.0)	346 (88.0)
Rural	13 (9.9)	34 (13.0)	47 (12.0)

*Marital Status*			
Married	113 (86.3)	236 (90.1)	349 (88.8)
Single	18 (13.7)	26 (9.9)	44 (11.2)

*Occupation*			
Peasants	20 (15.3)	54 (20.6)	74 (18.8)
No formal occupation	40 (30.5)	78 (30.0)	118 (30.1)
Petty trader	50 (38.2)	103 (39.3)	153 (38.9)
Employed	21 (16.0)	27 (10.3)	48 (12.2)

*Religion*			
Christian	102 (77.9)	200 (76.3)	302 (76.8)
Muslim	29 (22.1)	62 (23.7)	91 (23.2)

*Level of Education*			
None	10 (7.6)	11 (4.2)	21 (5.3)
Primary	74 (56.5)	161 (61.5)	235 (59.8)
Secondary	29 (22.1)	72 (27.5)	101 (25.7)
College	18 (13.8)	18 (6.9)	36 (9.2)

**Table 2:** Obstetric and clinical information of pregnant women enrolled.

Patient characteristics	Cases, number (%)	Controls, number (%)	All, number (%)
*Parity*			
Nulliparous	60 (45.8)	115 (43.9)	175 (44.5)
Para 2-3	56 (42.8)	112 (42.7)	168 (42.8)
≥ Para 4	15 (11.4)	35 (13.4)	50 (12.7)

*Gestation age*			
2^nd^ trimester	4 (3.0)	0 (0.0)	4 (1.0)
3^rd^ trimester	127 (97.0)	262 (100.0)	389 (99.0)

*Symptoms of UTI*			
Symptomatic UTI	11 (8.4)	4 (1.5)	15 (3.8)^*∗*^
Asymptomatic	120 (91.6)	258 (98.5)	378 (96.2

*Significant bacteriuria*			
Positive	66 (50.4)	44 (16.8)	110 (28.0)
Negative	65 (49.6)	218 (83.2)	283 (72.0)

*Blood pressure at booking*			
Mean SBP ± SD (mmHg)	115.0 ± 11.5	105.9 ± 10.6	109.2 ± 11.7
Mean DBP ± SD (mmHg)	71.3 ± 9.0	68.2 ± 7.6	69.4 ± 8.3

*Current blood pressure*			
Mean SBP ± SD (mmHg)	160.5 ± 16.2	116.0 ± 10.6	130.0 ± 24.5
Mean DBP ± SD (mmHg)	104.6 ± 11.0	72.1 ± 9.3	83.0 ± 18.2

*Preeclampsia severity*			
Non severe	62 (47.3)	NA	62 (47.3)
Severe	69 (52.7)	NA	69 (52.7)

*HIV serostatus*			
Negative	130 (99.2)	259 (98.9)	389 (99.0
Positive	1 (0.8)	3 (1.1)	4 (1.0)

^**∗**^Dysuria, suprapubic pain, fever, or pyuria; SBP: systolic blood pressure, DBP: diastolic blood pressure, SD: standard deviation, and NA: not applicable.

**Table 3:** Conditional logistic regression analysis for variables' association with preeclampsia.

Characteristics	OR	95% CI	P value
Hospital	0.65	0.36-1.16	0.143
Level of education	1.11	0.83-1.48	0.502
Occupation	1.22	0.96-1.56	0.100
Religion	0.91	0.54-1.53	0.726
Marital status	1.40	0.73-2.62	0.317
Parity	0.63	0.38-1.04	0.071
Gestation age	0.73	0.67-0.80	< 0.001
Significant bacteriuria	7.72	4.11-14.49	< 0.001

OR=odd ratio; CI=confidence interval.

**Table 4:** Antimicrobial resistance patterns of bacteria isolated from pregnant women.

Resistance n (%)	*Bacteria isolates*							
	*E. coli*		*Klebsiella* spp.		*Acinetobacter* spp.		Other GNB^*∗*^	
	Cases (N=31)	Controls (N=19)	Cases (N=15)	Controls (N=11)	Cases (N=4)	Controls (N=7)	Cases (N=12)	Controls (N=4)
AMP	30 (96.7)	18 (94.7)	15 100)	11 (100)	NA	NA	12 (100)	4 (100)
AMC	23 (74.2)	17 (89.5)	13 (86.7)	10 (90.9)	NA	NA	10 (83.3)	3 (75.0)
SXT	29 (93.6)	17 (87.5)	14 (93.3)	11 (100)	4 (100)	7 (100)	12 (100)	3 (75.0)
NF	7 (22.6)	4 (21.1)	9 (60.0)	7 (63.6)	NA	NA	7 (58.3)	2 (50.0)
GN	7 (22.6)	5 (26.3)	4 (26.7)	2 (18.20	1 (25.0)	2 (28.6)	6 (50.0)	3 (75.5)
CIP	3 (9.7)	5 (26.3)	5 (33.3)	3 (27.3)	1 (25.0)	2 (28.6)	3 (25.0)	1 (25.0)
CFZ	7 (22.6)	3 (15.8)	4 (26.7)	1 (9.1)	NA	NA	7 (58.3)	3 (75.0)
TZP	9 (29.0)	2 (10.5)	4 (26.7)	3 (27.3)	1 (25.0)	2 (28.6)	5 (41.7)	3 (75.0)
IMP	0 (0.0)	0 (0.0)	1 (6.7)	0 (0.0)	0 (0.0)	0 (0.0)	0 (0.0)	0 (0.0)

AMP: ampicillin; AMC: amoxycillin-clavulinate, SXT:trimethoprim-sulfamethoxazole, NF: nitrofurantoin; GN: gentamicin; CIP: ciprofloxacin; CFT: ceftriaxone; TZP: piperacillin- tazobactam; IMP: imipenem; GPB: Gram positive bacteria; GNB: Gram negative bacteria; *Klebsiella pneumoniae* (n=17); *Klebsiella oxytoca* (n=9); ^**∗**^[*Enterobacter aerogenes* (6)*, Citrobacter freundii *(1), *Proteus vulgaris (5); Proteus mirabilis (1); *Unidentified Gram negative Enterobacteriaceae (3)].

## Data Availability

The data used to support the findings of this study are available from the corresponding author upon request.

## Abbreviations

AST: Antimicrobial susceptibility testing
BMC: Bugando Medical Centre
ESBL: Extended spectrum beta lactamases
LMICs: Low and middle income countries
SRRH: Sekou Toure Regional Referral Hospital
UTIs: Urinary tract infections.
